# Clinical Use of the ImmunoCAP Inhibition Test in the Diagnosis of Meat Allergy Caused by a Tick Bite in an Adult Male with No Previous Atopic History

**DOI:** 10.3390/life13030699

**Published:** 2023-03-05

**Authors:** Kinga Lis, Natalia Ukleja-Sokołowska, Kornelia Karwowska, Joanna Wernik, Małgorzata Pawłowska, Zbigniew Bartuzi

**Affiliations:** 1 Department of Allergology, Clinical Immunology and Internal Medicine, Ludwik Rydygier Collegium Medicum in Bydgoszcz, Nicolaus Copernicus University in Toruń, ul. Ujejskiego 75, 85-168 Bydgoszcz, Poland; 2 Department of Infectious Diseases and Hepatology, Ludwik Rydygier Collegium Medicum in Bydgoszcz, Nicolaus Copernicus University in Toruń, ul. Sw. Floriana 12, 85-030 Bydgoszcz, Poland

**Keywords:** tick bite, α-GAL, mammalian meat allergy, cross-reactivity

## Abstract

(1) Background: alpha-gal syndrome (AGS) is a serious, potentially life-threatening allergic reaction. This is a type of food allergy to red meat and other mammalian products (e.g., gelatin). In Poland, this problem seems to be rare or, more likely, very underdiagnosed. The diagnosis of AGS is difficult. It seems that the knowledge about this syndrome is insufficient. There are no effective diagnostic tools able to clearly diagnose this cross-reactive allergy. This paper presents the clinical application of a non-standard method in the diagnosis of a cross-reactive allergy using the example of AGS. (2) Methods: standard tests for in vitro allergy diagnostics and the non-standard ImmunoCAP inhibition test(IT) were carried out for serum collected from a patient with a red meat allergy. (3) Results: the serum concentration of anti-α-Gal IgE was very high (302 kUA/L), and IgE antibodies toanti-mammalian-meat allergens were found. The level of IgE antibodies to mammalian meat allergens decreased after blocking on α-GAL-CAP. The concentration of anti-α-Gal IgE decreased after blocking on CAPs coated with various mammalian meat allergens. Blocking with allergens of poultry meat did not affect the concentration of anti-α-Gal IgE. (4) Conclusions: the ImmunoCAP ITseems to be a useful tool in the diagnosis of cross-reactive allergies. Based on their clinical history and test results, the patient was diagnosed with AGS caused by a primary sensitization to α-Gal after a tick bite. This is the second case of AGS described in Poland and the first in Pomerania.

## 1. Introduction

Alpha-gal syndrome (AGS) is a serious, potentially life-threatening allergic reaction. It is a type of food allergy to red meat and other products made from mammals. AGS is also called α-gal allergy, red meat allergy or tick bite meat allergy. It can manifest as an immediate drug allergy to pharmaceuticals containing α-Gal and a delayed hypersensitivity response to the ingestion of mammalian meat products [1]. The first references to an association between α-Gal allergy and red meat allergy appeared around 2006 and concerned unusual allergic reactions in people treated with Cetuximab [2,3]. Later, it was noticed that people who had been bitten by ticks previously manifested similar symptoms after ingestion of mammalian meat [4]. In all cases, the common factor was the presence of IgE antibodies to the carbohydrate determinant galactose-α-1,3-galactose, referred to as α-Gal [5].

The spectrum of AGS symptoms varies greatly. Usually, these include various types of allergic reactions, including skin lesions, digestive tract disorders, edema or anaphylaxis with varying degrees of delay, from several minutes to up to 6 h (according to most available studies, from 2 to 6 h) after eating a meal with meat. These cases often involve the presence of a cofactor in the form of exercise or simultaneous consumption of alcohol. It is also interesting that in some cases, despite the presence of IgE antibodies to α-Gal, patients without any symptoms of hypersensitivity can eat red meat [5,6,7,8,9,10,11,12].

The mechanism leading to AGS, especially with regard to the variability of the clinical symptoms and the delay in the response, is not fully understood. Various theories are taken into account, including environmental factors, the efficiency of the immune system and presentation via a different mechanism, and the kinetics of allergen digestion and absorption, especially with regard to animal fats. It is suspected that α-gal is absorbed on the surface of chylomicrons (as a glycolipid), and in this form, it is delivered to immunocompetent cells. This could indeed be responsible for the delay in the reaction, as fats are absorbed from the gastrointestinal tract particularly efficiently between 2 and 6 h after consumption, which explains the important role of alcohol as a cofactor in these reactions [11,13,14,15,16].

It seems that in Poland, AGS is a very rare phenomenon or its occurrence is very underdiagnosed. To date, only one case of AGS has been described in Poland [17]. There is also one population analysis of the occurrence of anti-α-Gal IgE antibodies in forest workers from the Podlasie region [18].

## 2. Materials and Methods

Laboratory tests were conducted for blood serum taken from a 65-year-old man who had several anaphylactic reactions after eating meat.

There were three stages in this study:

- Patient’s clinical history—detailed analysis;
- Standard laboratory methods;
- Non-standard laboratory method—ImmunoCAP inhibition test (IT).

### 2.1. Patient’s Clinical History—Detailed Analysis

The patient’s standard clinical history and detailed personal allergic history were examined. His atopic status was assessed. Detailed questions were asked about repeated anaphylaxis occurrences and the circumstances in which they occurred.

### 2.2. Standard Laboratory Methods

Extended laboratory diagnostics were performed according to the data from the patient’s clinical history. Laboratory tests routinely used for in vitro allergy diagnosis were carried out:

- ALEX2—multiplex allergy test (Allergy Xplorer, MacroArray Diagnostics; MADx);
- Specific IgE—for the allergens: α-Gal, extracts of mammalian meat allergens (beef, pork, mutton, rabbit), beef gelatin and poultry meat allergens (chicken, turkey)—ImmunoCAP (Thermo Fisher Scientific, Waltham, MA, USA);
- Total IgE—ImmunoCAP (Thermo Fisher Scientific, Waltham, MA, USA).

The ALEX2 (Allergy Xplorer, MacroArray Diagnostics; MADx) is a nano-bead technology, ELISA (enzyme-linked immunosorbent assay) protocol-based IgE multiplex assay. The ALEX2 is a quantitative specific IgE and semi-quantitative total IgE test. The ALEX2 test simultaneously measures serum concentrations of IgE specific for 117 allergen extracts and for 178 allergen components and total IgE. The lowest detectable level of IgE in the test was 0.1 kUA/L. Specific IgE results are presented in classes 0–4, where class 0 (<0.3 kUA/L; negative or borderline), class 1 (0.3–1 kUA/L; low concentration), class 2 (1–5 kUA/L; moderate concentration), class 3 (5–15 kUA/L; high concentration) and class 4 (>15 kUA/L; very high concentration). The manual Image Xplorer system with Raptor software was used in the experiment.

ImmunoCAP (Thermo Fisher Scientific, Waltham, MA, USA) is an in vitro test system for the quantitative measurement of specific IgE and total IgE in human serum or plasma via an ELISA-based fluoro-immuno-enzymatic method (FEIA). Specific IgE results are presented in classes 0–6, where class 0 (0.1 kUA/L; undetectable and 0.1–0.34; detectable-low), class 1 (0.35–0.69 kUA/L; low), class 2 (0.7–3.49 kUA/L; moderate), class 3 (3.5–17.49 kUA/L; high), class 4 (17.5–49 kUA/L; very high), class 5 (50–99 kUA/L; very high) and class 6 (>100 kUA/L; very high). The Phadia100 system was used in the experiment.

### 2.3. Non-Standard Laboratory Method—ImmunoCAP Inhibition Test (IT)

**Figure 1:**
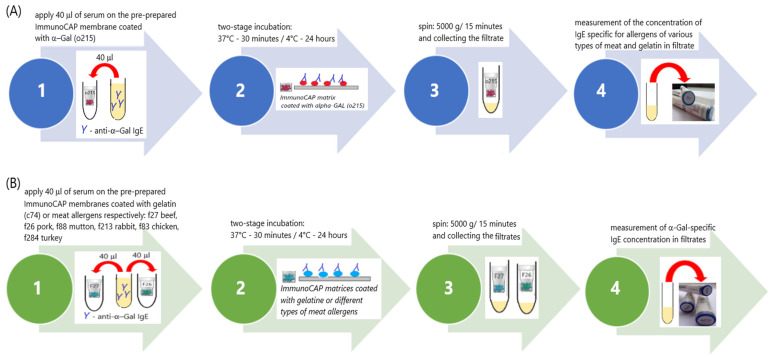
Scheme of the ImmunoCAP inhibition test (IT) (**A**) with α-Gal; (**B**) with gelatin or meat allergens (f27 beef, f26 pork, f88 mutton, f213 rabbit, f83 chicken, f284 turkey).

Experimental ImmunoCAP inhibition tests (ITs) were performed according to the procedures designed in our laboratory, the schemes for which are shown in Figure 1A,B.

In the experiment, we used “CAPs”, which are components of the ImmunoCAP system. CAPs are the carriers of the allergen. They are a plastic container in which a cellulose matrix coated with allergens is placed. This was the solid phase of the test—the allergen carrier.

The following types of CAPs were used for the tests:

- Scheme A: o215-CAP (α-Gal; bovine thyroglobulin—TBG);
- Scheme B: f27-CAP (beef), f26-CAP (pork), f88-CAP (mutton), f213-CAP (rabbit), f83-CAP (chicken), f284-CAP (turkey) and c74-CAP (beef gelatin).

The first stage of both schemes was identical. At this stage, solid-phase CAPs were prepared for binding specific IgE antibodies from the patient’s serum with the appropriate allergens coated on the CAPs. Each CAP was placed inside dry, clean Eppendorf tubes (volume 1500 µL). Then, 40 µL of washing solution was added to each CAP. The washing solution that is part of the ImmunoCAP system (Art. No. 10-9422-01) was used. The solution was prepared according to the manufacturer’s instructions. All CAPs sealed inside Eppendorf tubes were centrifuged (5000× *g*; 15 min). The CAPs were transferred to clean, dry Eppendorf (volume 1500 µL) tubes and the filtrate was discarded. In this way, 9 o215-CAPs and 2 CAPs, each from a different type of CAP used later on in the experiment, were prepared. The number of CAPs needed was determined considering the volume of serum needed for further analysis. The prepared CAPs were used in the next stage of the experiment.

The next stage of the experiment was divided into two branches: Scheme A (Figure 1A) and Scheme B (Figure 1B).

***Branch A (Figure 1A)***

Prepared o215-CAPs were used in this branch of the experiment. A total of 40 µL of patient serum was applied to each CAP. CAPs tightly sealed in Eppendorf tubes were incubated in two stages: first for 30 min at 37 °C and then for 24 h at 2–8 °C. After the incubation, the CAPs were centrifuged (5000× *g*; 15 min). After centrifugation, the CAPs were discarded, and the filtrates were collected in a single tube. The IgE concentration anti-α-Gal, anti-f27 (beef), anti-f26 (pork), anti-f88 (mutton), anti-f213 (rabbit), anti-f83 (chicken), anti-f284 (turkey) and anti-c74 (beef gelatin) were measured in the filtrate, using the ImmunoCAP system with Phadia100.

***Branch B (Figure 1B)***

In this branch of the experiment, the other prepared CAPs (f27-CAP, f26-CAP, f88-CAP, f213-CAP, f83-CAP, f284-CAP and c74-CAP) were used. A total of 40 µL of patient serum was applied to each CAP. CAPs tightly sealed in Eppendorf tubes were incubated in two stages: first for 30 min at 37 °C and then for 24 h at 2–8 °C. After the incubation, the CAPs were centrifuged (5000× *g*; 15 min). After centrifugation, the CAPs were discarded, and the filtrates were collected. The filtrates from the same types of CAPs were combined in one tube. Altogether, 7 types of filtrates were obtained. The concentration of anti-α-Gal IgE (ImmunoCAP, Phadia100) was measured in each of them.

## 3. Results

### 3.1. Patient’s Clinical History—Detailed Analysis—Results

Based on the patient’s clinical history, it was found that the first severe allergic reaction of the immediate type occurred in January 2010. Prior to this episode of anaphylaxis, the patient had not experienced any allergic reactions. He was a healthy, non-atopic man without any chronic diseases. During this first episode of anaphylaxis (in January 2010), the patient developed edema and hives of the scalp, hips and anus, combined with severe pruritus. There was also a rapid fall in blood pressure and loss of consciousness. Specialized treatment in the emergency department was necessary. Subsequent episodes of allergic reactions with a similar course occurred in the patient in September 2010, autumn 2014, December 2017 and several times from July 2019 to January 2020.

During this period, there were also several reactions of a lesser severity, which were not accompanied by loss of consciousness, and the spectrum of symptoms was limited to swelling, hives and itching of the previously mentioned body areas.

In turn, during the last three episodes, the previously observed symptoms of a severe systemic reaction were additionally joined by swelling of the lips and edema and urticarial changes in the hands.

According to the patient, both the frequency and severity of the symptoms had increased recently. Severe systemic reactions always required treatment in a hospital emergency department. For less severe episodes limited to urticaria and pruritus, short-term treatment with low-dose antihistamines or oral steroids was sufficient.

The man could not clearly identify the common cause of the allergic reactions. He associated them with the consumption of small volumes of various types of alcohol or meals with meat. He also drew attention to the possible contribution of physical effort related to practicing sports (such as cycling or skiing).

The clinical history of the patient, the spectrum of observed symptoms after the meals with meat, the presence of allergic reaction cofactors (alcohol, physical exertion) and the absence of features of atopic disease before the first episode of severe allergic reaction raised the suspicion of α-Gal syndrome (AGS).

To verify our hypothesis, we extended the clinical interview by asking the patient whether it was likely that he had been bitten by a tick in the time preceding the first episode of acute allergic reaction. The patient confirmed being bitten by a tick in July 2009. It happened near the Tri-City (Pomerania, Poland), when he was cycling to work in the morning. The tick was removed and tested for Lyme disease (the tick was not a carrier). Unfortunately, the patient did not have the result of this test and was unable to determine the species of tick that bit him.

After obtaining this information, we proposed the patient perform additional laboratory tests, verifying our suspicions as to whether he was allergic to the polysaccharide, α-Gal, found in tick saliva, and the related possibility of severe allergic reactions after eating mammalian meat, especially in the presence of the cofactor.

### 3.2. Standard Laboratory Methods—Results

In the first stage of standard laboratory diagnostics for allergic disease, the ALEX2 multiparameter test was performed. The ALEX2 test showed a very high concentration of total IgE. A low concentration of IgE antibodies specific for airborne and food allergens of plant origin as well as hymenoptera venom allergens were detected. A moderate level of IgE antibodies specific for all mammalian meat allergens represented in the ALEX2 test were detected. No IgE antibodies to poultry meat allergens were found. No specific IgE antibodies for the allergen components important in the diagnosis of primary allergy to meat, Bos d 6 and Sus d 1, were detected. IgE results specific for other allergens represented in the ALEX 2 test were negative. The ALEX2 results are presented in Table 1.

The ImmunoCAP method confirmed a very high total IgE concentration. The IgE antibody anti-α-Gal was detected at a very high concentration. The concentration of IgE antibodies for beef allergen extract was very high, as well as that for pork allergen extract. A moderate level of IgE antibodies to mutton and rabbit meat allergens were found. IgE antibodies to poultry meat allergens (chicken, turkey) and beef gelatin allergens were not detected. The anti-α-Gal IgE to total IgE ratio was 11.61%. All results of these analyses are presented in Table 2.

### 3.3. Non-Standard Laboratory Method—ImmunoCAP Inhibition Test (IT)—Results

**Figure 2:**
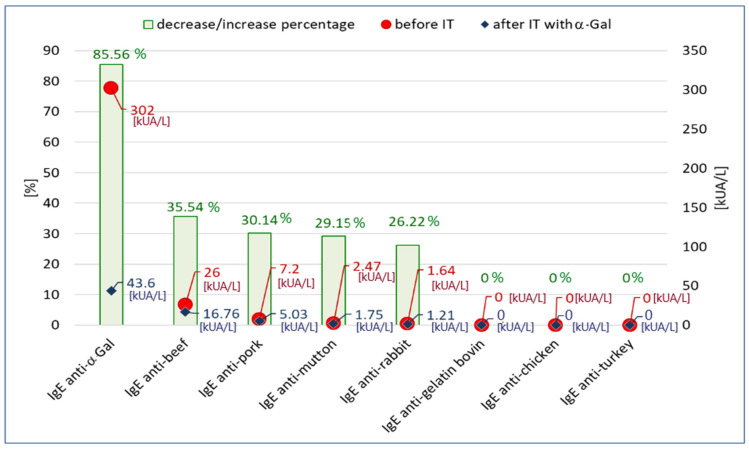
The concentration of specific IgE antibodies specific to α-Gal, meat allergens and bovine gelatin before the inhibition test (IT) and after inhibition on o215-CAP and the percentage change of concentrations.

***Branch A (Figure 2)***

In branch A of the ImmunoCAP inhibition test (Figure 1A), we assumed that if the reactivity to meat allergens was due to the presence of anti-α-Gal IgE antibodies, the removal of these antibodies from the serum would cause it to not bind meat allergens. This means that the concentration of IgE specific for meat allergens after the inhibition test would be lower than before the test or undetectable.

The results of the inhibition test on o215-CAP (α-Gal-coated CAP; α-Gal-CAP) are shown in Figure 2. We observed that as a result of incubation of the patient’s serum on α-Gal-CAP, the concentration of anti-α-Gal IgE decreased by 85.56% (this confirms that o215-CAP binds anti-α-Gal antibodies). The reduction in IgE specific for mammalian meat allergens (beef, pork, mutton, rabbit) ranged from 35.54% to 26.22%. Specific IgE concentrations for gelatin and poultry meat (chicken, turkey) were negative (<0.1 kUA/L) both before and after the inhibition test. In the experiment, a 93.85% decrease in total IgE concentration was also observed as a result of the inhibition (2602 kU/L vs. 160 kU/L).

***Branch B (Figure 2)***

In branch B of the ImmunoCAP inhibition test (Figure 1B), we assumed that if meat allergens express the α-Gal polysaccharide on their surface, they bind anti-α-Gal antibodies from the patient’s serum. The inhibition of anti-α-Gal antibodies should result in a decrease in their concentration in the serum after the inhibition test on CAPs coated with allergens of various meats.

**Figure 3:**
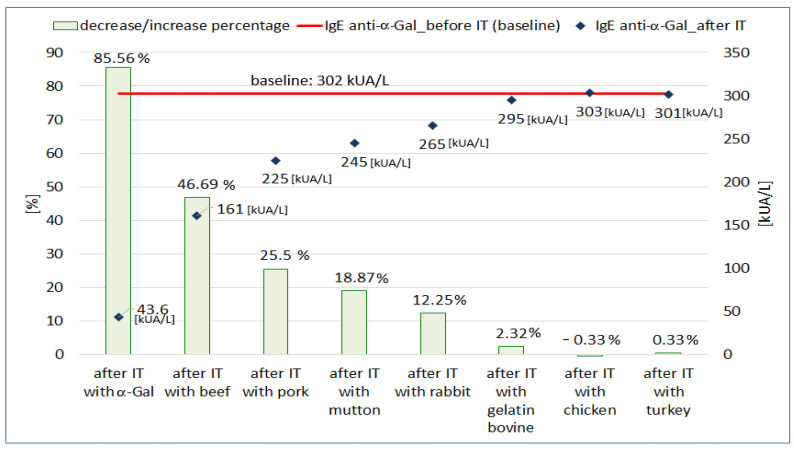
The concentration of IgE antibodies specific to α-Gal before the inhibition test (IT) and after inhibition on CAPs with α-Gal, allergens of various types of meat or bovine gelatin and the percentage change of concentrations.

The results of the inhibition test on membranes coated with various meat or gelatin allergens are shown in Figure 3. In this experiment, we observed a decrease in the concentration of anti-α-Gal IgE antibodies after incubation on CAPs coated with mammalian meat allergens (beef, pork, mutton, rabbit). The concentration of anti-α-Gal IgE decreased from 46.69% to 12.25%. Incubation on beef-gelatin-coated CAPs reduced anti-α-Gal IgE antibody concentrations by 2.32% compared to baseline. When the CAPs were coated with poultry allergens, only minimal, bi-directional changes in anti-α-Gal IgE concentration were observed (−0.33% for chicken and 0.33% for turkey). The mean level of total IgE after incubation on CAPs coated with mammalian meats allergens was 1758 kU/L and decreased by 31.62% compared to the initial value (2571 kU/L). After incubation on gelatin-coated CAP, the decrease in total IgE concentration was only 0.51% (2558 kU/L vs. 2571 kU/L). Incubation of the patient’s serum on CAPs coated with poultry allergens did not change the total IgE concentration compared to the pre-incubation value (2570 kU/L vs. 2571 kU/L).

### 3.4. Summary of Diagnostic Results

**Figure 4:**
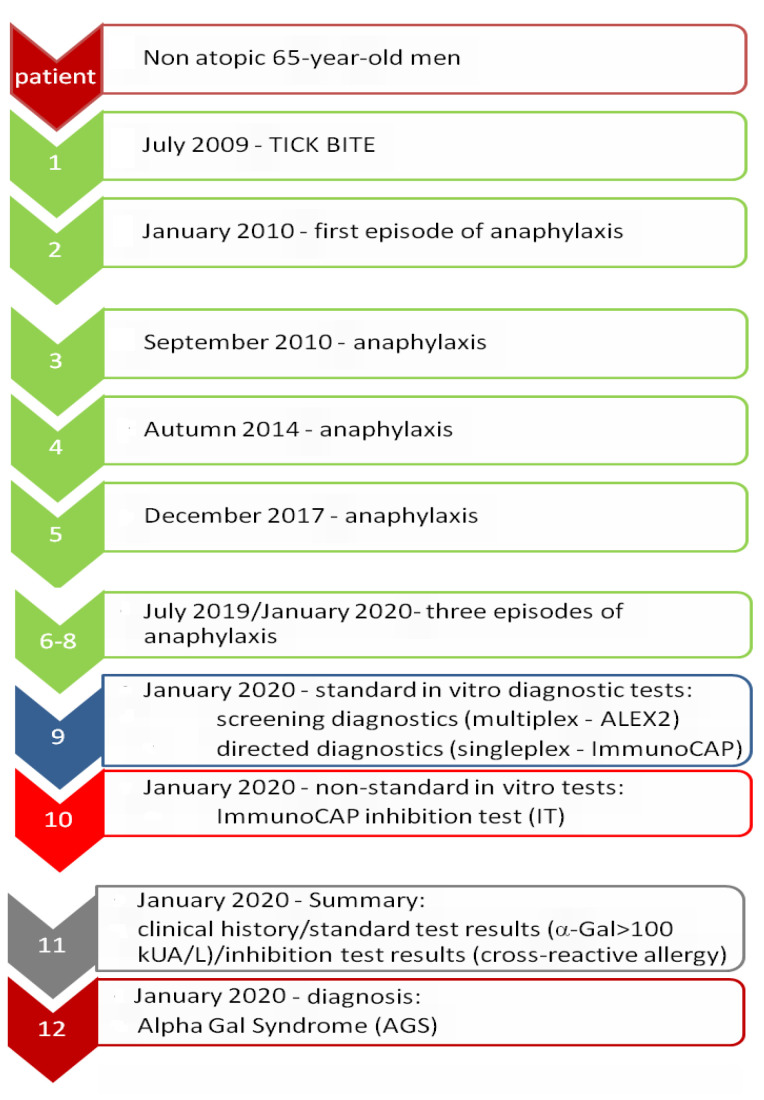
Sequence of clinical symptoms from tick bite to diagnosis of alpha-gal syndrome in a previously non-atopic 65-year-old man.

The patient’s clinical history, sequence of symptoms and subsequent steps of laboratory diagnosis are summarized in Figure 4. The diagram shows the suggested location for the proposed experimental ImmunoCAP inhibition tests in the diagnostic process.

## 4. Discussion

AGS is a group of symptoms of a severe allergic reaction to mammalian meat and meat-derived products (e.g., gelatin, biological drugs) which consists of an allergy to the determinant of the carbohydrate α-Gal derived from tick saliva. Primary sensitization develops after a tick bite, and the reaction to meat is associated with the cross-reactivity of anti-α-Gal IgE antibodies with the α-Gal expressed on mammalian meat proteins [1,5].

AGS was originally thought to be endemic and found mainly in the Southeastern United States. However, there are currently case reports of AGS from all continents [19]. It seems that in Poland, tick-bite-related meat allergy is a rare clinical syndrome. Currently, only two studies on this subject are available [17,18]. However, it is not known whether this is really a rare syndrome of clinical symptoms or whether it is simply rarely recognized.

A characteristic clinical feature of AGS is the occurrence of symptoms of severe allergy after eating red meat. It is characteristic that symptoms do not appear immediately after a meat meal, but are delayed for several hours. The presence of a cofactor is also often necessary. The reaction can also develop in people who were not previously allergic [5,7,8,9,10,11,12].

We described a patient who developed a severe allergic reaction a few hours after eating meals with meat, a case which fits perfectly into the area of symptoms of AGS. This 65-year-old man has never suffered from any symptoms of an allergic disease. Only after being bitten by a tick 10 years ago did he develop his first episode of anaphylaxis. It seems, therefore, that the tick bite itself was the factor that initiated these events.

It is also significant that the presence of anaphylaxis cofactors often distracts the patient from the true cause of these reactions. In the described case, the man assumed that the cause of his anaphylaxis was the consumption of various types of alcohol. Only the demonstration of the presence of antibodies to mammalian meat allergens and the connection of this result with the clinical history of the patient, supplemented with information about the tick bite, raised the suspicion of AGS in this patient.

The quantitative determination of IgE α-Gal antibodies has been successfully used in the diagnosis of AGS. According to Fischer et al. [20] an anti-α-Gal IgE concentration ≥0.54 kUA/L is the optimal cut-off point for assessing the diagnostic value of alpha-gal syndrome in allergic patients. In turn, according to Mabelane et al. [21], the values best qualifying allergy to α-Gal are an anti-α-Gal IgE level of 2.00 kU/L and a ratio of specific IgE anti-α-Gal/total IgE of at least 0.75%. Platts-Mills et al. [5] report that if the level of IgE antibodies specific to α-Gal is ≥2 IU/mL or more than 2% of total IgE, an allergic reaction after eating red meat is very likely. Our patient met all these criteria. His serum concentration anti-α-Gal IgE was very high (302 kUA/L) and his anti-α-Gal IgE/total IgE ratio was 11.6%.

Mammalian meat is the most common trigger for allergic reactions in patients with AGS [1,5]. In our patient’s serum, IgE antibodies specific for beef, pork, mutton and rabbit meat were present. It was the presence of these antibodies in the absence of IgE antibodies specific to poultry meat allergens and antibodies to serum albumin (Bos d 6 and Sus d 1) that raised the suspicion of AGS in the described case. Beef and beef offal are mentioned by some authors as the main meat sources causing symptoms of AGS [22,23,24]. Our results seem to confirm these suggestions. In addition, in the ImmunoCAP inhibition test on α-Gal-coated CAP, the percentage decrease in the concentration of antibodies specific for beef was higher than for meat of other mammals. Additionally, beef significantly reduced the concentration of anti-α-Gal IgE antibodies in the inhibition test on CAPs coated with allergens of various mammalian meats. These observations seem to confirm the important role of beef in causing AGS symptoms.

It is assumed that α-Gal IgE may also be a target for antibodies in gelatin allergy [5,25]. However, we did not find gelatin-specific IgE antibodies in our patient. It is also worth noting that gelatin very weakly blocked anti-α-Gal IgE antibodies in the ImmunoCAP inhibition test (c74-CAP). Perhaps the method of obtaining gelatin and its technological processing are important for the exposure of α-Gal in gelatin products.

Diagnostics of AGS is a difficult and multi-stage process. It often requires combining seemingly unrelated facts and laboratory results. The first signal that led to the suspicion of alpha-gal syndrome in the described patient was the detection, using the ALEX2 test, of the presence of IgE antibodies to mammalian meat allergens in the serum. The ALEX2 test is a multiparameter screening test used in the diagnosis of allergies. The results of these types of tests often need to be confirmed by reference methods according to the clinical symptoms observed in the patient. Based on the patient’s clinical history, we confirmed the ALEX2 results by measured the concentration of specific IgE antibodies to meat allergens using a reference test (ImmunoCAP), and we determined the concentration of anti-α-Gal IgE antibodies because we suspected that AGS was the cause of our patient’s recurrent anaphylaxis. After detecting antibodies to α-Gal in the patient serum, we decided to check whether the presence of antibodies to meat was caused only by cross-reactivity to α-Gal expressed on meat allergens. Since there are no standard tests to study this type of relationship, we performed an experimental inhibition test on factory-allergen-coated CAPs, which is an element of the ImmunoCAP system. We chose these substrates because of their properties. They are usually used to determine the concentration of specific IgE antibodies in the routine diagnosis of allergic diseases. The allergens that are used to coat the ImmunoCAP CAPs are obtained by the manufacturer of the ImmunoCAP reagents under standardized conditions. The manufacturer guarantees the stability and purity of these extracts; thus, the results are repeatable. We assumed that they would be a very good source of well-characterized allergens. The experiments showed that α-Gal antigens bind anti-α-Gal IgE antibodies and IgE antibodies specific for mammalian meat allergens, reducing their serum concentration. Additionally, allergens of various mammalian meats bind anti-α-Gal IgE antibodies, reducing their serum concentration. These results seem to confirm that the cause of the symptoms observed in the described patient is not a primary allergy to meat, but a cross-allergy caused by sensitization to α-Gal that developed following a tick bite.

Due to the variability of clinical manifestations, it seems that many difficult or unclear cases of meat allergy remain undiagnosed or misdiagnosed. This may have a significant impact on the therapeutic and dietary management, the quality of the patient’s life and his safety [5]. The diagnosis of alpha-gal syndrome is relatively late. Flaherty et al. [6] estimated that in the group of patients they studied, the average time from the onset of AGS symptoms to diagnosis was over 7 years. It is worth noting that in our patient, 11 years had passed since the tick bite in July 2009 until the diagnosis of AGS in December 2020. Additionally, the first episode of anaphylaxis requiring intervention in the emergency department occurred in January 2010, that is, 6 months after the tick bite and 10 years before the diagnosis of AGS.

The presented clinical case study is an example of the clinical application of the experimental ImmunoCAP inhibition test, the procedure for which was designed by us. Inhibition tests are non-standard analytical methods used in scientific research to study the cross-reactivity of allergens [26,27,28,29,30,31]. Allergies are often associated with cross-reactivity of antibodies directed to epitopes of similar structure found in different allergen sources. Unfortunately, there are still no standardized tools useful in the routine diagnosis of allergies based on such a pathophysiological mechanism. Various models of inhibition tests have already been successfully used in scientific research by both our team [26,27,28] and other researchers [29,30,31]. We have previously successfully used inhibition tests in the diagnosis of anaphylaxis sensitization to mango [26] and sunflower seed [27], which resulted from a cross-reaction in mugwort-allergic patients. Both in our opinion and in the opinion of other authors [30,31], all inhibition tests used to study the phenomenon of cross-reactivity can be both a valuable tool for the study of cross-reactivity between allergens and a useful tool in the diagnosis of allergies resulting from these reactions. Knowledge of the amino acid sequence and antigen conformation is not always sufficient to predict the true reactivity of allergens with specific antibodies under natural conditions.

## 5. Conclusions

The results of laboratory analyses, both standard and experimental tests, combined with the patient’s clinical symptoms, allowed us to establish a logical sequence that ultimately led to the development of AGS as a result of sensitization to tick saliva polysaccharides in a primarily non-atopic adult male. It seems that the experimental inhibition ImmunoCAP test used in this study may be a valuable diagnostic tool for testing cross-reactivity of allergens in clinical practice.

We advised our patient to exclude mammal meat from his diet and replace it with poultry meat, which is safe in the case of alpha-gal syndrome. Due to his history of anaphylactic reactions, the patient was informed that he should always carry a syringe pre-filled with adrenaline and was trained in its use. The patient is compliant with the dietary recommendations and has had no further episodes of anaphylaxis.

## 6. Study Limitations and Future Perspectives

The main limitation of the presented study is that only the results obtained for one patient were used to assess the clinical utility of the proposed ImmunoCAP inhibition test (IT). It seems, however, that this test may be a valuable tool in the study of the clinical significance of allergens cross-reactivity, especially if we take into account that we currently do not have any other standardized diagnostic tools in this area. By presenting this description, we want to encourage other researchers to undertake similar activities and search for new, unconventional applications of commonly available laboratory tools.

## Figures and Tables

**Table 1:** The ALEX2 test results (specific IgE results for other allergens were negative).

Total IgE (tIgE)(kU/L): 2602							
Specific IgE (airborne allergens—pollens)(kUA/L):							
Aln g 1 (alder pollen; PR-10 protein)			Amb a (ragweed pollen; extract)		Amb a 1 (ragweed pollen; pectate lyase)		
0.49			0.47		0.40		
Specific IgE (plant food allergens) (kUA/L):							
Act d 2 (kiwi; thaumatin-like protein; TLP)				Mal d 1 (apple; PR-10 protein)			
0.62				0.44			
Specific IgE (animal food allergens—extracts) (kUA/L):							
Bos d meat (beef)	Sus d meat (pork)		Equ c meat (horse- flesh)	Ovi a meat (lamb/ mutton)	Ory c meat (rabbit meat)	Gal d meat (chicken meat)	Mel g meat (turkey meat)
2.03	0.38		1.8	<0.1	0.76	<0.1	<0.1
Specific IgE (animal food allergens—molecules) (kUA/L):							
Bosd 6 (serum albumin)				Sus d 1 (serum albumin)			
<0.1				<0.1			
Specific IgE (Hymenoptera insect venom allergens) (kUA/L):							
Api m (honey bee venom; extract)		Ves v (wasp venom; extract)		Ves v 5 (wasp venom; Antigen 5)			
0.31		0.57		1.31			

**Table 2:** The ImmunoCAP results.

Specific IgE(kUA/L):							
**o215** (α-Gal) *	f27 (beef)	f26 (pork)	f88 (mutton)	f213 (rabbit)	c74 (gelatin bovine)	f83 (chicken)	f284 (turkey)
302	26.0	7.20	2.47	1.64	<0.1	<0.1	<0.1
Total IgE (tIgE) (kU/L): 2571							
anti-α-Gal IgE/total IgE ratio (%): 11.61							

Remarks: * The anti-α-Gal sIgE concentration in the serum was higher than 100 kUA/L (>100 kUA/L). Due to the detection limit of the ImmunoCAP method for sIgE, which is 100 kUA/L, the serum was diluted 1:10 to measure the final concentration of anti-α-Gal IgE.

## Data Availability

Not applicable.

## References

[1] Avila S.A., Wojno T. (2022). Alpha-Gal Syndrome: A New Etiology for Periorbital Edema. Ophthalmic Plast Reconstr. Surg..

[2] Chung C.H., Mirakhur B., Chan E., Le Q.T., Berlin J., Morse M., Murphy B.A., Satinover S.M., Hosen J., Mauro D. (2008). Cetuximab-induced anaphylaxis and IgE specific for galac-tose-alpha-1,3-galactose. N. Engl. J. Med..

[3] Lammerts van Bueren J.J., Rispens T., Verploegen S., van der Palen-Merkus T., Stapel S., Workman L.J., James H., van Ber-kel P.H., van de Winkel J.G., Platts-Mills T.A. (2011). Anti-galactose-α-1,3-galactose IgE from allergic patients does not bind α-galactosylated glycans on intact therapeutic antibody Fc domains. Nat. Biotechnol..

[4] Commins S.P., Satinover S.M., Hosen J., Mozena J., Borish L., Lewis B.D., Woodfolk J.A., Platts-Mills T.A. (2009). Delayed ana-phylaxis, angioedema, or urticaria after consumption of red meat in patients with IgE antibodies specific for galac-tose-alpha-1,3-galactose. J. Allergy Clin. Immunol..

[5] Platts-Mills T.A.E., Li R.C., Keshavarz B., Smith A.R., Wilson J.M. (2020). Diagnosis and Management of Patients with the α-Gal Syndrome. J. Allergy Clin. Immunol. Pract..

[6] Flaherty M.G., Kaplan S.J., Jerath M.R. (2017). Diagnosis of Life-Threatening Alpha-Gal Food Allergy Appears to Be Patient Driven. J. Prim. Care Community Health.

[7] Jappe U., Minge S., Kreft B., Ludwig A., Przybilla B., Walker A., Varga R., Seidel P., Biedermann T., Anemüller W. (2018). Meat allergy associated with galactosyl-α-(1,3)-galactose (α-Gal)-Closing diagnostic gaps by anti-α-Gal IgE immune profiling. Allergy.

[8] Ün M., Dağaşan S. (2020). Alpha-Gal Syndrome. JEB Med. Sci..

[9] Richards N.E., Richards R.D. (2021). Alpha-Gal Allergy as a Cause of Intestinal Symptoms in a Gastroenterology Community Practice. South Med. J..

[10] Wilson J.M., Schuyler A.J., Schroeder N., Platts-Mills T.A. (2017). Galactose-α-1,3-Galactose: Atypical Food Allergen or Model IgE Hypersensitivity?. Curr. Allergy Asthma Rep..

[11] Mateo Borrega M.B., Garcia B., Larramendi C.H., Azofra J., González Mancebo E., Alvarado M.I., Alonso Díaz de Durana M.D., NúñezOrjales R., Diéguez M.C., Guilarte M. (2019). IgE-Mediated Sensitization to Galactose-α-1,3- Galactose (α-Gal) in Urticaria and Anaphylaxis in Spain: Geographical Variations and Risk Factors. J. Investig. Allergol. Clin. Immunol..

[12] Mabelane T., Ogunbanjo G.A. (2019). Ingestion of mammalian meat and alpha-gal allergy: Clinical relevance in primary care. Afr. J. Prim. Health Care Fam. Med..

[13] Chakrapani N., Fischer J., Swiontek K., Codreanu-Morel F., Hannachi F., Morisset M., Mugemana C., Bulaev D., Blank S., Bindslev-Jensen C. (2022). α-Gal present on both glycolipids and glycoproteins contributes to immune response in meat-allergic patients. J. Allergy Clin. Immunol..

[14] Iweala O.I., Choudhary S.K., Addison C.T., Batty C.J., Kapita C.M., Amelio C., Schuyler A.J., Deng S., Bachelder E.M., Ainslie K.M. (2020). Glycolipid-mediated basophil activation in alpha-gal allergy. J. Allergy Clin. Immunol..

[15] Levin M., Apostolovic D., Biedermann T., Commins S.P., Iweala O.I., Platts-Mills TA E., Savi E., van Hage M., Wilson J.M. (2019). Galactose α-1,3-galactose phenotypes: Lessons from various patient populations. Ann. Allergy Asthma Immunol..

[16] Iweala O.I., Choudhary S.K., Addison C.T., Commins S.P. (2021). T and B Lymphocyte Transcriptional States Differentiate between Sensitized and Unsensitized Individuals in Alpha-Gal Syndrome. Int. J. Mol. Sci..

[17] Brzozowska M., Mokrzycka N., Porebski G. (2021). Alpha-gal syndrome: The first report in Poland. Cent. Eur. J. Immunol..

[18] Rutkowski K., Sowa P., Mroczko B., Pancewicz S., Rutkowski R., Czupryna P., Groblewska M., Łukaszewicz-Zając M., Mo-niuszko-Malinowska A. (2022). Sensitisation and allergic reactions to alpha-1,3-galactose in Podlasie, Poland, an area endemic for tick-borne infections. Infect. Dis..

[19] Boyce R.M., Schulz A., Mansour O., Giandomenico D., Farel C.E., Commins S.P. (2022). Alpha-Gal Syndrome in the Infectious Diseases Clinic: A Series of 5 Cases in Central North Carolina. Open Forum. Infect. Dis..

[20] Fischer J., Huynh H.N., Hebsaker J., Forchhammer S., Yazdi A.S. (2020). Prevalence and Impact of Type I Sensitization to Alpha-Gal in Patients Consulting an Allergy Unit. Int. Arch. Allergy Immunol..

[21] Mabelane T., Basera W., Botha M., Thomas H.F., Ramjith J., Levin M.E. (2018). Predictive values of alpha-gal IgE levels and alpha-gal IgE: Total IgE ratio and oral food challenge-proven meat allergy in a population with a high prevalence of reported red meat allergy. Pediatr. Allergy Immunol..

[22] Villalta D., Cecchi L., Farsi A., Chiarini F., Minale P., Voltolini S., Scala E., Quercia O., Muratore L., Pravettoni V. (2017). Galactose-α-1,3-galactose syndrome: An Italian survey. Eur. Ann. Allergy Clin. Immunol..

[23] Popescu F.D., Vieru M. (2018). Precision medicine allergy immunoassay methods for assessing immunoglobulin E sensitization to aeroallergen molecules. World J. Methodol..

[24] Pisazka V., Duscher G., Hodžić A., Reider N., Allerberger F. (2019). Alpha-gal allergy after a tick bite in Austria. Wien Klin Wochenschr..

[25] Mullins R.J., James H., Platts-Mills T.A., Commins S. (2012). Relationship between red meat allergy and sensitization to gelatin and galactose-α-1,3-galactose. J. Allergy Clin. Immunol..

[26] Ukleja-Sokołowska N., Gawrońska-Ukleja E., Lis K., Żbikowska-Gotz M., Sokołowski Ł., Bartuzi Z. (2018). Anaphylactic reaction in patient allergic to mango. Allergy Asthma Clin. Immunol..

[27] Ukleja-Sokołowska N., Gawrońska-Ukleja E., Żbikowska-Gotz M., Bartuzi Z., Sokołowski Ł. (2016). Sunflower seed allergy. Int. J. Immunopathol. Pharmacol..

[28] Lis K., Ukleja-Sokołowska N., Adamczak R., Bartuzi Z. (2022). Experimental Research Models to Assess the Cross-Reactivity between Can f 5 and Human PSA-Two Different Perspectives. Int. J. Mol. Sci..

[29] Sweeney M.J., Kay C., Klotz L.R., Klotz S.D. (1981). An IgE inhibition assay for the detection of allergen specific IgE. Ann. Allergy.

[30] Schmidt-Hieltjes Y., Teodorowicz M., Jansen A., den Hartog G., Elfvering-Berendsen L., de Jong N.W., Savelkoul H.F., Ruinemans-Koerts J. (2017). An alternative inhibition method for determining cross-reactive allergens. Clin. Chem. Lab. Med..

[31] Bastiaan-Net S., Batstra M.R., Aazamy N., Savelkoul HF J., van der Valk JP M., Gerth van Wijk R., Schreurs MW J., Wichers H.J., de Jong N.W. (2020). IgE cross-reactivity measurement of cashew nut, hazelnut and peanut using a novel IMMULITE inhibition method. Clin. Chem. Lab. Med..

